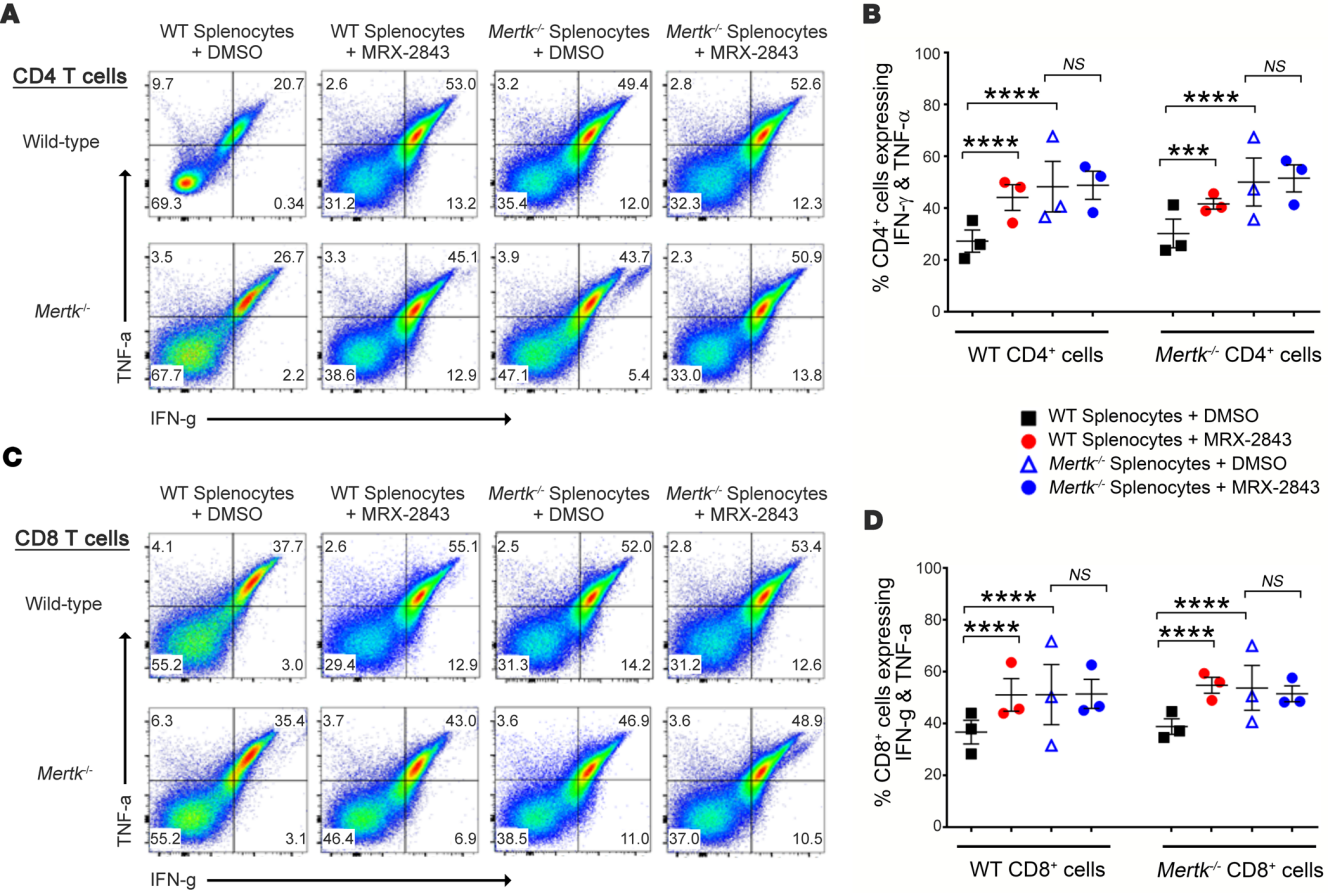# MERTK inhibition alters the PD-1 axis and promotes anti-leukemia immunity

**DOI:** 10.1172/jci.insight.145847

**Published:** 2020-12-03

**Authors:** Alisa B. Lee-Sherick, Kristen M. Jacobsen, Curtis J. Henry, Madeline G. Huey, Rebecca E. Parker, Lauren S. Page, Amanda A. Hill, Xiaodong Wang, Stephen V. Frye, H. Shelton Earp, Craig T. Jordan, Deborah DeRyckere, Douglas K. Graham

Original citation: *JCI Insight*. 2018;3(21):e97941. https://doi.org/10.1172/jci.insight.97941

Citation for this corrigendum: *JCI Insight*. 2020;5(23):e145847. https://doi.org/10.1172/jci.insight.145847

The graph shown in Figure 6D was inadvertently duplicated in Figure 6B. However, the data shown in Figure 6 were correctly analyzed and statistics remain as published. The corrected Figure 6 is below. The article has been updated with the corrected information.

The authors regret the error.

## Figures and Tables

**Figure 6:**